# Wall Slip Behaviour of Polymers Based on Molecular Dynamics at the Micro/Nanoscale and Its Effect on Interface Thermal Resistance

**DOI:** 10.3390/polym12102182

**Published:** 2020-09-24

**Authors:** Yan Lou, Gang Wu, Yanfeng Feng

**Affiliations:** Guangdong Provincial Key Laboratory of Micro/Nano Optomechatronics Engineering, College of Mechatronics and Control Engineering, Shenzhen University, Shenzhen 518060, China; wugang4637@163.com (G.W.); yffeng0209@szu.edu.cn (Y.F.)

**Keywords:** molecular dynamics, micro/nanoscale, polymer, wall slip, interface thermal resistance

## Abstract

Taking the Poiseuille flow of a molten polymer in parallel plates as the research object and polymethyl methacrylate (PMMA) as the research material, an all-atom analysis model of the molecular dynamic flow of polymer macromolecules is established according to the Navier slip law. The effects of wall wettability and external pressure on the wall slip behaviour of polymer macromolecules, as well as the spatial evolution process of the entanglement–unentanglement process of polymer chains near the wall under different shearing effects, were studied. The interface thermal resistance rule was explored, and an interface thermal resistance model considering the wall slip behaviour was established. Finally, a micro-injection experiment was used to verify the validity and accuracy of the model. The results show that when the wall is hydrophobic, the polymer melt exhibits significant wall slip. As the external pressure increases, the wall slip speed and the slip length increase. However, after a certain pressure is exceeded, the growth rate of the slip length is basically zero. As the external pressure increases, the PMMA molecular chains gradually start to separate, the single molecular chain becomes untangled from the entangled grid, and the chain detaches from the wall after exceeding a certain threshold. Wall slip reduces the interface thermal resistance between the solid–liquid interface and enhances the interface heat transfer performance. The interface thermal resistance value calculated by molecular dynamics can more accurately reflect the heat conduction rule of the solid–liquid interface at the micro/nanoscale than that measured by the thermal resistance experiment, indicating that the micro/nano interface thermal resistance obtained by molecular dynamics simulation is reliable.

## 1. Introduction

Micro/nano-injection moulding increases the surface-to-body ratio of micro/nanoscale cavities, which intensifies the heat transfer at the solid–liquid interface and leads to rapid cooling of a melt. Wall slip and thermal contact resistance (TCR) are important boundary parameters in numerical simulations, and they determine the accuracy of micro/nano-injection moulding simulations.

Many scholars have begun to use molecular dynamics (MD) simulations to study the wall slip phenomenon. For example, Martini et al. [1] used MD to simulate the wall slip length and found that there is a threshold at a higher shear rate; the slip length does not change after exceeding this value. Bendada et al. [2] reported that in the early stage of injection moulding, polymer macromolecular chains produced tensile orientation deformation under the action of flow-induced stress. In the later stage of mould filling, as the orientation deformation time was prolonged, the molecular chains unwrapped due to the orientation deformation time. The knots tended to be dispersed. At the same time, it is considered that there is a stress threshold. When the stress is greater than this value, the molecules loosen, and untangling occurs. Sridhar [3] used MD to simulate the injection process of polymethyl methacrylate (PMMA) nanopillars and found that PMMA molecules stretched along the sidewalls and were highly oriented along the flow direction.

Second, the interface TCR is mainly obtained through experiments and theoretical derivation. Hong et al. [4] calculated the TCR through recursive methods based on indirectly measuring the filling height of patterns as a function of time, and estimated the changes in the TCR with time and position. Dawson et al. [5] experimentally determined that the maximum heat transfer coefficient between the melt of PMMA material and the surface of the cavity is 7000 W/m^2^ K and introduced the concept of an air layer; that is, after the melt is filled, cooled and allowed to contract, there is an air layer between the wall and the plastic part in the cavity. The interface heat transfer coefficient (HTC; the reciprocal of the TCR) in the presence of the air layer was studied, and a relevant model was established. Some et al. [6] established a rough interface TCR model that simultaneously correlates pressure and temperature and found that the model can reliably predict TCR even during polymer crystallisation at the contact interface. Liu et al. [7] focused on evaluating the HTC between the polymer and the cavity wall and established a mathematical model to improve the simulation of cooling and crystallinity development, thereby improving the accuracy of injection moulding simulations to predict the mechanical properties and dimensional accuracy of injection-moulded parts. Zhou et al. [8] analysed the relationship between TCR and cooling time by developing a three-dimensional transient cooling analysis module for injection moulds, and they constructed a TCR calculation model. Wang et al. [9] found that during rapid thermal cycle formation, the heat transfer coefficient increased sharply in the initial stage, gradually decreased and finally reached the equilibrium state; the larger the Reynolds number is, the higher the heat transfer coefficient.

However, few scholars have performed studies to simulate the TCR of polymer walls based on MD. He [10] and Arduini-Schuster et al. [11] conducted MD simulations of the heat transfer of aerogel and foam microstructures, respectively, but only studied the heat transfer rules without external pressure. Song et al. [12] used MD simulations to study the effect of wall slip on the interface thermal resistance for liquid argon and found that the wall slip at low flow rates had no significant effect on the interface thermal resistance, while the wall slip at high flow rates would cause the interface thermal resistance to be reduced by approximately 10%; however, the simulations only involved a liquid with a simple molecular chain and did not consider a polymer macromolecular chain.

In this study, the Poiseuille flow of a molten polymer between parallel plates was used as the research object and PMMA was used as the research material. According to the Navier slip law, an all-atom analysis model of polymer macromolecules is established. By studying the effect of wall wettability and external driving pressure on the wall slip behaviour of polymer macromolecules and the spatial evolution process of the entanglement–unentanglement of the polymer chain near the wall under different shearing effects, the interface thermal resistance rule at the micro/nanoscale was elucidated. The influence of wall slip on the heat conduction under plastic processing pressure of polymer was also revealed, and the interface thermal resistance model considering the wall slip was established. The effectiveness and accuracy of the model were verified by micro-injection experiments. The difference between simulation results and experimental measurements was due to the wall surface being set to an ideal smooth state in the simulation process. In the following work, a nanoscale fractal rough surface model is established using fractal theory and molecular dynamics, to obtain more accurate simulation results.

## 2. PMMA Polymer Molecular Dynamics Model

### 2.1. Model Construction

The polymer molecular dynamics model is constructed with Materials Studio (2017 edition, Accelrys Software Inc., San Diego, CA, USA) and the open source molecular dynamics calculation program LAMMPS (Large-scale Atomic/Molecular Massively Parallel Simulator) [13]. The simulation calculation platform is a Dell 7820 graphics workstation with 2.2 GHz CPU frequency (Dell Computer Corporation, Austin, Texas, USA). The model is mainly composed of a molten polymer and a metal mould.

**Figure 1:**
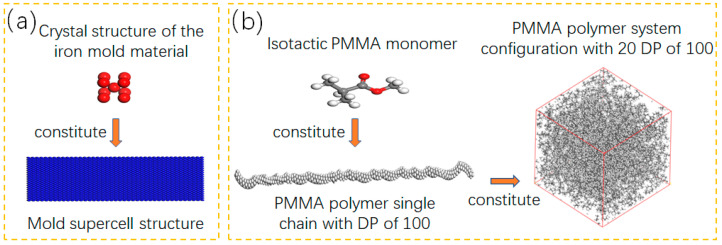
Construction of the polymethyl methacrylate (PMMA) polymer molecular dynamics model: (**a**) Mold supercell structure; (**b**) PMMA polymer system configuration with 20 DP of 100.

First, the body-centred cubic unit cell of the iron mould material is established as shown in Figure 1a, where the red part represents the crystal structure of iron, and the blue plate represents the supercell structure obtained by modelling based on the crystal structure of iron (red part). Second, according to the structural characteristics of PMMA materials, the required molecular structure is established by the visualiser function in Materials Studio. Because of the differences in polymerisation mechanisms, PMMA has three different structural manifestations: isotactic, syndiotactic and atactic. In practical industrial applications, isotactic PMMA is widely used, so isotactic PMMA is used as the initial monomer structure (Figure 1b). Finally, using this structural monomer as the smallest repeating unit, a single-chain polymer structure with a degree of polymerisation (DP) of 100 is established by the build module in Materials Studio. Then, the amorphous cell tool is used to build an amorphous polymer system with 20 single chains of the PMMA polymer with a DP of 100, as shown in Figure 1b.

To obtain a reasonable and stable initial conformation, it is necessary to optimise the structure and minimise the energy to obtain a stable single-chain polymer. The initial temperature of the polymer is set to 300 K, the density is 1.18 g/cm^3^, the size of the simulated system is 10 nm × 10 nm × 10 nm, and the channel width of the polymer melt region is 5 nm. The configuration parameters of the PMMA polymer system are shown in Table 1.

The system performs 10^3^ iterations by the Forcite dynamics calculation module in Materials Studio, and the energy convergence level is set to 1 × 10^−5^ kcal/mol. The smart method was used to optimise the geometric structure. Then, the energy is relaxed by an annealing treatment, and the annealing simulation is performed for 5 cycles by the anneal function in the Forcite module so that the energy of the system is stable. There is a total of 5 cycle periods, and a single cycle gradient is 50 K. The temperature change range is 200 to 500 K, and a single cycle gradient is calculated at 1000 steps. A total of 5 × 10^5^ steps are calculated at the end of 5 cycle periods.

**Figure 2:**
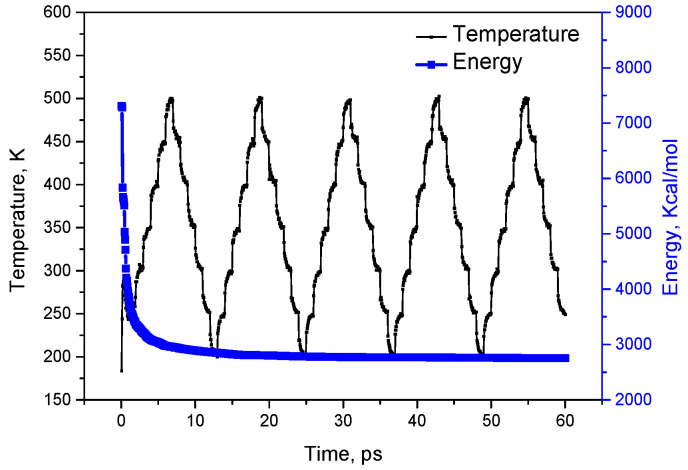
The change curves of system temperature and energy during the entire annealing process.

Figure 2 shows the system temperature and energy changes during the entire annealing calculation. After five annealing simulation calculations, the system energy is reduced from 7300 kcal/mol to approximately 3000 kcal/mol and stabilised. The five annealing cycles were used to calculate the energy that can balance the amorphous system. After the annealing calculation is completed, a total of five trajectories are obtained, and the fifth frame trajectory is extracted as the initial conformation for the next polymer flow calculation.

**Figure 3:**
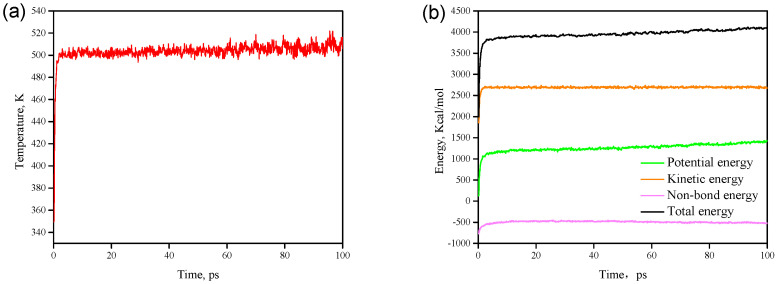
PMMA system relaxation process: (**a**) change curve of temperature; (**b**) change curve of energy.

The PMMA material architecture obtained after geometric optimisation and energy minimisation must also be subject to relaxation treatment. Figure 3 shows the temperature and energy change curves during relaxation. It can be clearly seen that after a 100 ps relaxation treatment, the fluctuation range of the temperature is within ±10 K (Figure 3a), and the energy fluctuation is within 5% (Figure 3b), indicating that the polymer is at equilibrium at this time.

### 2.2. Pressure Parameter Setting

**Figure 4:**
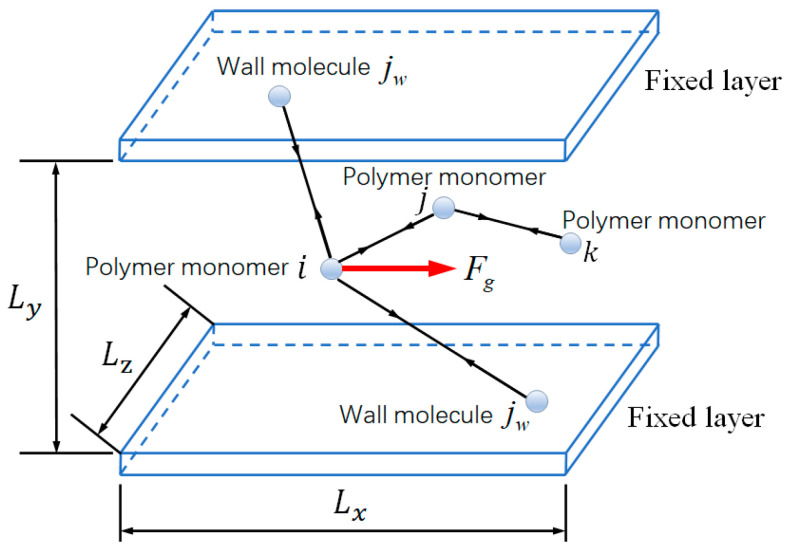
Force analysis of polymer flow.

The upper and lower plates are fixed in the calculation process, and the polymer melt in the middle is affected by the $F_{g}$ (Figure 4). There are two main sources of force in the polymer chain: one is the force exerted by the external pressure $F_{g}$, and the other is the force $F_{i}$ between the particles of the polymer chain itself and the LJ potential.

Then, the force acting on the polymer monomer is

(1) $$F=F_{g}+F_{i}+\frac{\partial \Phi_{LJ}}{\partial r_{ij}}+\frac{\partial \Phi_{wf\;-\;LJ}}{\partial r_{ij_{w}}}$$

where $F_{g}$ is the force exerted by the external pressure, $F_{i}$ is the interaction force between polymer monomers, $\Phi_{LJ}$ is the potential energy function between monomers, $r_{ij}$ is the position vector between monomers i and j, $\Phi_{wf\;-\;LJ}$ is the potential function of monomer i and wall $j_{w}$ and $r_{ij_{w}}$ is the position vector of single i and wall $j_{w}$.

The interaction force between polymer monomers is calculated as $F_{i}=-\frac{\partial \Phi_{F}}{\partial r_{i}}$, where $\Phi_{F}\left(r\right)=-1/2H_{s}r_{0}{}^{2}\ln[1\;-\;\frac{r_{i}^{2}}{r_{0}{}^{2}}]$, $H_{s}$ is the elastic modulus, $r_{i}$ is the unit length and $r_{0}$ is the maximum elongation of the monomer polymer chain.

For Poiseuille fluid flow, under the action of external pressure $F_{g}$, there are two effects: (a) driving fluid flow and (b) conversion into heat, which increases the temperature of the fluid. In the simulation calculation process, the temperature must be controlled to prevent an unrestricted increase in the fluid temperature, so the wall temperature control method is used in this study.

### 2.3. Model Layered Statistics

The model of polymer fluid flow between parallel plates generally stratifies the entire *y*-axis direction of the fluid into Y layers and creates a function of $G_{n}\left(y_{ij}\right)$,

(2) $$G_{n}(y_{ij})=\{\begin{array}{cc} 1 & (n-1)\Delta y<y_{ij}<n\Delta y\\ 0 & y_{ij}\leq (n-1)\Delta y,y_{ij}\geq n\Delta y \end{array}$$

where $y_{ij}$ represents the position of the particle i at the jth step. Then, Equations (3)–(5) can be used to calculate the density ρ, velocity v and temperature T of the single-layer particles.

(3) $$\rho_{n}\sigma^{3}=\frac{\sigma^{3}}{L_{x}L_{z}\Delta y(J_{m}-J_{n}+1)}\sum_{j=J_{n}}^{J_{m}}\sum_{i=1}^{N}G_{n}(y_{ij})$$

(4) $$v\left(y_{ij}\right)=\frac{1}{\left(J_{m}\;-\;J_{n}+1\right)\sum_{i=1}^{N}G_{n}\left(y_{ij}\right)}\overset{J_{m}}{\underset{j=J_{n}}{\sum }}\overset{N}{\underset{i=1}{\sum }}G_{n}\left(y_{ij}\right)v_{ij}$$

(5) $$T(y_{ij})=\frac{1}{3k_{B}(J_{m}-J_{n}+1)\sum_{i=1}^{N}G_{n}(y_{ij})}\sum_{j=J_{n}}^{J_{m}}\sum_{i=1}^{N}G_{n}(y_{ij})m_{i}{\left[v_{i,j}^{a}-u^{a}\right]}^{2}$$

## 3. Experiments

### 3.1. Experiment to Measure the Interface TCR

To obtain the TCR value between the solid–liquid interface, it is necessary to measure the mould surface temperature and the melt temperature. The heat flux density through the interface is calculated by the temperature recorded by the thermocouple. The platform to measure the interface TCR includes a micro-injection moulding machine (Babyplast 6/10, Rambaldi Group, Montoro, Italy), an injection mould (homemade), a thermocouple (TT-K-36-SLE, Omega Engineering Inc., Norwalk, CT, USA) and a real-time data acquisition system (NI USB-9162, National Instruments, Austin, TX, USA).

**Figure 5:**
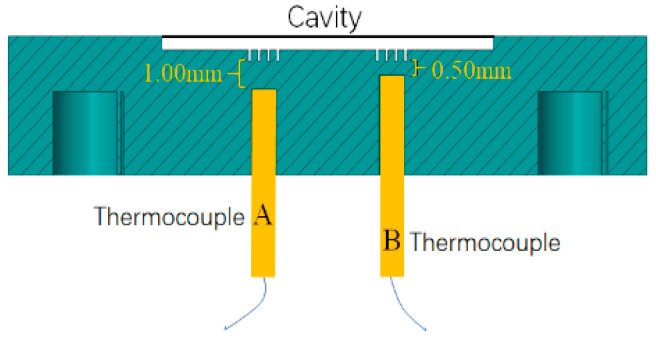
Schematic diagram of the thermocouple layout.

The mould core contains inserted blocks to install the temperature sensor, and the installation position of the temperature sensor in the mould core is shown in Figure 5. Two thermocouples were placed at the same time by drilling holes in the mould core. The installation positions of the two thermocouples are parallel to each other inside the mould core and are perpendicular to the mould parting surface. The distances from the lower surface of the microstructure cavity are 0.50 and 1.00 mm. The sensors are connected to the data acquisition card, which can measure the real-time internal temperature of the mould during the micro-injection moulding process. For reliable results, under each set of process parameters, the temperature was measured repeatedly 5 times, and then the average value was taken for analysis and calculation.

According to heat transfer theory, during the micro-injection moulding process, the high-temperature melt enters the mould cavity under the action of a certain injection pressure, and the heat is first transferred to the mould cavity wall surface and then transferred to the mould through the solid–liquid interface. Finally, the heat diffuses into the air. Therefore, it can be approximated that the heat flux interacting between the polymer and the wall surface is equal to the heat flux flowing through the mould. The temperature of the wall can be measured by the sensor to obtain the heat flux in the mould.

According to Newton’s law of cooling, the heat flux density $q_{1}$, that is, the amount of heat passing through a unit area per unit time, can be expressed by Equation (6),

(6) $$q_{1}=k\times \frac{T_{1}-T_{2}}{\Delta l}$$

where $q_{1}$ is the heat flux density in the mould, k is the mould material thermal conductivity, *T*_1_ and *T*_2_ are the temperatures measured by two temperature sensors at different positions and Δl is the distance between two temperature sensors in the thickness direction of the cavity.

Newton’s cooling law (Equation (7)) is used to calculate the heat transfer performance at the solid–liquid interface:

(7) $$q_{2}=h\times (T_{b}-T_{w})$$

where $q_{2}$ is the heat flux density of the molten polymer to the cavity wall, h is the heat transfer coefficient between the molten polymer and the cavity wall, $T_{b}$ is the temperature of the polymer melt and $T_{w}$ is the temperature of the cavity wall.

The temperature of the cavity wall can be expressed by Equation (8).

(8) $$T_{w}=T_{1}+(T_{1}-T_{2})$$

For $q_{1}=q_{2}$, the expression for calculating the convective heat transfer coefficient (Equation (9)) can be obtained:

(9) $$h=\frac{q_{1}}{T_{b}-T_{w}}$$

Then, the equation for the interface TCR (Equation (10)) is obtained.

(10) $$\mathrm{TCR}=\frac{T_{b}\;-\;T_{w}}{q_{1}}$$

### 3.2. Micro-Injection Moulding Experiments

Micro-injection moulding is performed using a micro-precision injection moulding machine (Babyplast 6/10, Rambaldi Group, Montoro, Italy). Its plunger diameter is 10 mm, the maximum clamping force per unit area is 15 MPa, the maximum injection volume is 15 mL, the total power is 3 kW, the maximum injection pressure is 15 MPa and the maximum injection speed is 80 mm/s.

PMMA material (IG-840, LG Group, Seoul, Korea) is used, and its basic properties are a thermal conductivity of 0.123 W/(m^2^ k), a specific heat capacity of 1717 kJ/(kg·°C), a melting point of 240 °C and a density of 1.0606 g/cm^3^. The length × width × thickness of the injection sample is 10 mm × 0.4 mm × 0.1 mm.

During the experiment, the injection pressure was set to 4.5, 6.5, 8.5, 11.5 and 13.5 MPa; the injection speed was 55 mm/s; and the injection temperature was 250 °C. To obtain a more accurate sample length, 5 samples were selected under each group of parameters in this study for averaging the data.

### 3.3. Finite Element Simulation of Micro-Injection Moulding

The finite element simulation uses Moldflow software (2017 edition, Autodesk Inc., San Rafael, CA, USA), and the injection moulding materials and process parameters are consistent with the actual injection experiments in Section 3.2. In the simulation, the double-layer mesh type is selected for mesh division, and the total number of meshes is 45,305. To compare the effectiveness of the interface TCR obtained by various methods, the interface TCR used in the simulation was selected based on the TCR value from the MD simulation, the default TCR value in Moldflow software, and the experimental TCR value from the interface thermal resistance measurement.

## 4. Results and analysis

### 4.1. Effect of Wall Wettability on Wall Slip

**Figure 6:**
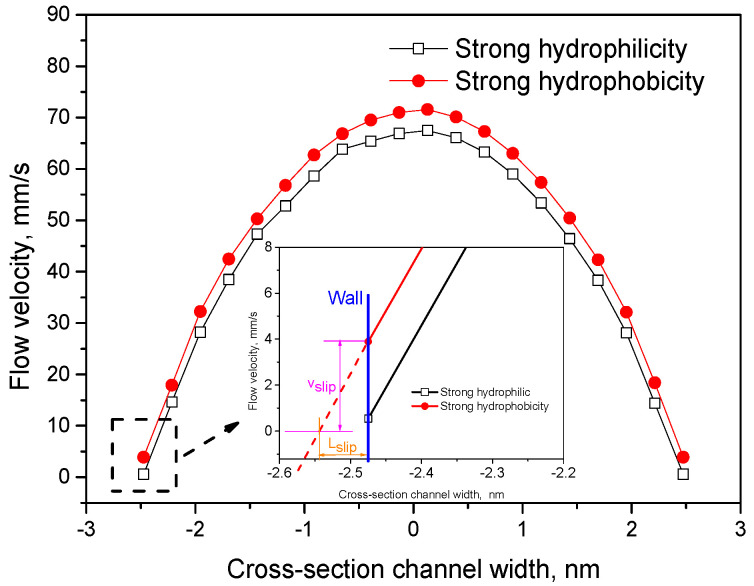
Velocity curve under different wetting conditions at external pressure $F_{g}=2\;\mathrm{MPa}$.

First, two wettability conditions under $F_{g}=2\;\mathrm{MPa}$ are studied. In this study, the two wettabilities are simulated by adjusting the interaction strength of the liquid–solid interface [12], and the strengths $\varepsilon_{1}=3.0\times 10^{-21}J$ and $\varepsilon_{2}=0.1\times 10^{-21}J$ indicate a strong hydrophilic wall and strong hydrophobic wall, respectively. The velocity distribution curve of the polymer melt in the channel in Figure 6 is obtained. It can be seen that when the wall is strongly hydrophobic ($\varepsilon_{2}=0.1\times 10^{-21}\;J$), the wall slip speed $V_{slip}=4\frac{\mathrm{mm}}{s}>0$. When the wall is strongly hydrophilic ($\varepsilon_{1}=3.0\times 10^{-21}\;J$), the wall slip speed $V_{slip}$ is approximately equal to zero, meaning that no slip occurs.

.

$V_{slip}$ is zero mainly because when the molten polymer passes through the strong hydrophilic wall, the hydrophilic group in the PMMA molecular chain adsorbs onto the metal atoms of the wall with a strong interaction force. When the external pressure is small, it is not strong enough to push the polymer molecular chain near the wall surface to overcome the adsorption energy and break away from the wall surface, which eventually leads to the formation of an adhesion layer at the solid–liquid interface. All the flow velocities of the polymer melt near the wall surface tend toward zero. In addition, $L_{slip}$ is the slip length of the polymer (Figure 6), which refers to the distance between the interface where no slip occurs and the actual metal interface.

### 4.2. The Effect of External Pressure on the Wall slip

The Navier slip model, $V_{slip}=L_{slip}\frac{\partial v_{x}}{\partial z}$, is used to explore the change rule of external pressure on the wall slip speed and slip length at the micro/nanoscale, where $V_{slip}$ represents the wall slip speed, $L_{slip}$ represents the slip length, $v_{x}$ represents the velocity of the polymer melt at the liquid-solid interface in the tangential direction and z represents the distance at the liquid–solid interface in the normal direction.

**Figure 7:**
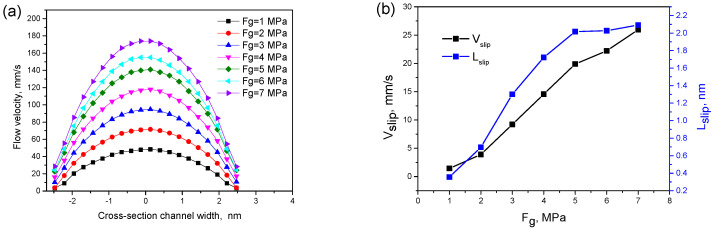
(**a**) Flow velocity curves under different external pressures; (**b**) wall slip speed and length under different external pressures.

Figure 7a shows the flow velocity curve of the polymer melt under different external pressures $F_{g}$ when the wall surface is strongly hydrophobic. It can be seen that the velocity distribution curves in the channel are nearly parabolic, which is symmetrical along the centreline (x = 0 nm), and there is obvious slip velocity at the boundary of the two sidewalls ($V_{slip}\neq 0$).

Figure 7b shows the wall slip speed and slip length under different external pressures. As $F_{g}$ increases, both $V_{slip}$ and $L_{slip}$ increase. At the same time, there is an external pressure threshold. When the external pressure $F_{g}>5\;\mathrm{MPa}$, the growth rate of the slip length $L_{slip}$ is approximately 0, but the wall slip speed $V_{slip}$ still increases. This is mainly due to the increase of external pressure and shear rate, which lead to the increase of hydrophobic wall slip velocity $V_{slip}$ and slip length $L_{slip}$. When the external pressure is greater than 5 MPa, the polymer chain completely detaches from the wall resulting in $L_{slip}$ remaining unchanged. However, slip velocity $V_{slip}$ is not affected by the detachment of the molecular chain from the wall and keeps increasing continuously.

### 4.3. Effect of External Pressure on Polymer Density

**Figure 8:**
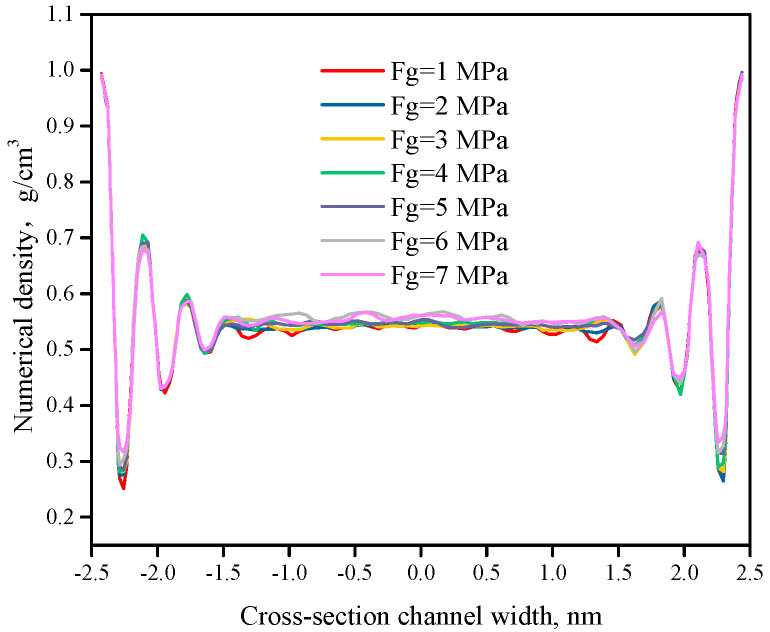
Numerical density curve of the polymer under different external pressures.

Figure 8 shows the numerical density curve of the polymer under different external pressures, revealing the spatial distribution of the polymer melt in the flow field. There is obvious polymer aggregation at the boundary positions (x = −2.5 nm) and (x = 2.5 nm) of the two sidewalls. When the external pressure increases from 1 MPa to 7 MPa, there is no obvious change in the curve profile, and the jump cycle of the density distribution remains the same. This indicates that the density distribution of the polymer melt is independent of the external pressure.

### 4.4. Influence of External Pressure on the Molecular Chain Morphology

**Figure 9:**
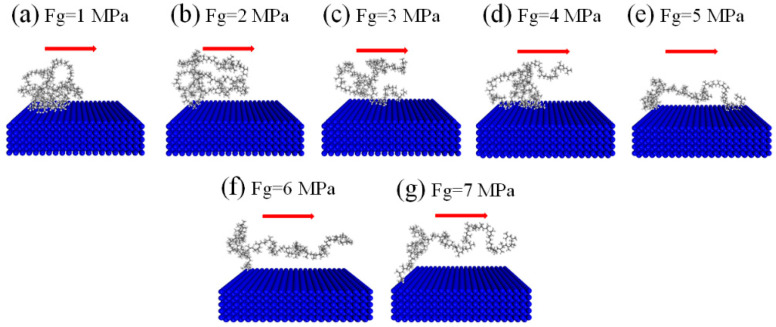
Change in the PMMA molecular chain morphology near the wall under different external pressures.

Figure 9 is a diagram of the change in the PMMA molecular chain morphology near the wall under different external pressures. To observe the change in the molecular chain morphology, only one PMMA molecular chain is shown. When the external pressure is 1 MPa, the polymer molecular chain is in a coiled state. When the external pressure gradually increases, the molecular chain begins to open. As the external pressure increases, the shear stress on the molecular chain increases, and the molecular chain overcomes the steric hindrance and increases the kinetic activation energy. Therefore, the more obvious the expansion is, the better the orientation is in the flow direction, which is consistent with the results presented by Jiang [14]. At the same time, that there is a driving pressure threshold of $F_{g}$ = 6 MPa; that is, when this threshold is reached, the polymer segment adhering to the wall finally detaches from the wall surface (Figure 9f).

### 4.5. Effect of Wall Slip on Interface TCR

**Figure 10:**
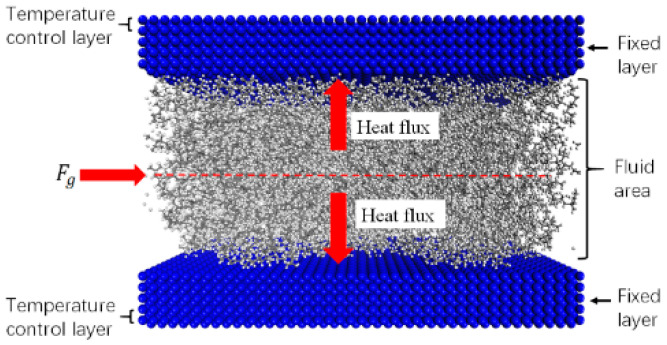
Polymer flow heat transfer model.

To calculate the interface TCR using MD methods, first, the atomic structure of the wall is fixed, and the penultimate layer of atoms on the wall is set as the temperature control area to keep it away from the solid–liquid interface to prevent the temperature from affecting the interface (Figure 10). Second, different external pressures are applied to atoms in the fluid area to drive them to flow in the channel. Finally, the heat flow, q, and the interface thermal resistance, $\mathrm{TCR}=\frac{\Delta T}{q}$, are calculated.

**Figure 11:**
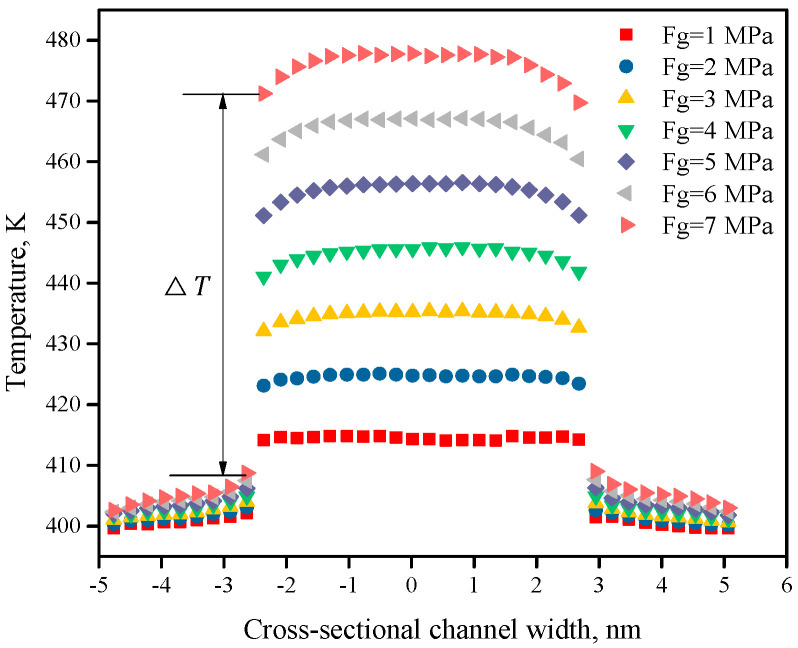
Temperature jump at the interface under different external pressures.

The interface TCR at the micro/nanoscale causes a temperature jump at the solid–liquid interface, resulting in boundary conditions that are different from the macroscopic characteristic scale. Figure 11 shows the temperature jump at the interface under different external pressures. When the external pressure is increased from 1 to 7 MPa, the temperature on the walls increases slightly with the external pressure. However, the temperature in the central area increases obviously with increasing driving pressure; that is, the wall temperature jump ΔT increases. The main reason is that the increasing external pressure causes the polymer to be subjected to increasingly stronger shearing. The temperature of the polymer chain naturally increases due to shearing, so the temperature in the central area increases, but due to the interface thermal resistance at the solid–liquid interface, a significant temperature jump occurs at the interface.

**Figure 12:**
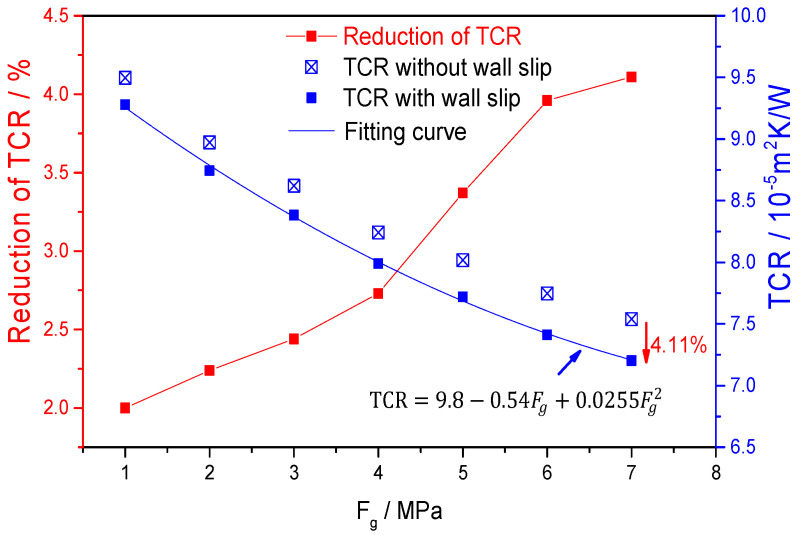
Thermal contact resistance (TCR) values under different external pressures.

Figure 12 shows the change in the interface thermal resistance under different external pressures. As the external pressure increases, the wall slip and the temperature of the polymer melt both increase. This study mainly explores the effect of the wall slip on the interface thermal resistance, so the interface thermal resistance under the same external pressure with and without wall slip is compared (Figure 12).

As the external pressure increases, the interface TCR decreases more with wall slip than without wall slip. When the external pressure is 7 MPa, the TCR with wall slip is smaller than without wall slip, which reaches 4.11%. Therefore, it can be considered that the wall slip at the micro/nanoscale has an effect on the interface thermal resistance. The main reason is that as the external pressure increases, the wall slip speed increases (Figure 7), which in turn enhances the molecular vibration, and the energy transfer mainly depends on the molecular vibration. Therefore, increasing the driving pressure can increase the energy transfer at the solid–liquid interface and reduce the interface thermal resistance. At the same time, the wall slip of the polymer does not have a significant effect on the interface thermal resistance. The reason is that, on the one hand, the wall slip caused by the external pressure did not affect the density distribution of the polymer melt at the interface (Figure 8). As the energy exchange at the solid–liquid interface is mainly related to the thermal vibration of the interface normal, the wall slip mainly affects the tangential movement of the melt [15]. Finally, based on the interface TCR with wall slip in Figure 12, the model of interface TCR is established by polynomial fitting, as shown in Equation (11).

(11) TCR/(10−5m2K/W)=9.8−0.54(Fg/MPa)+0.0255(Fg/MPa)^2

### 4.6. Verification of the Micro-Injection Experiment

**Figure 13:**
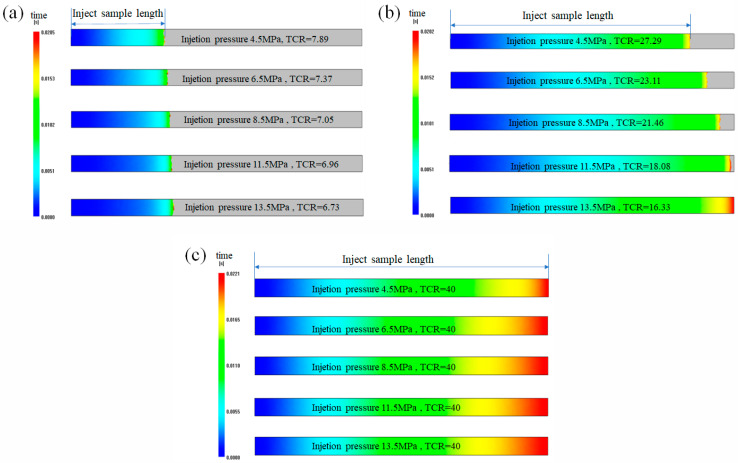
Moldflow simulation of injection sample length: TCR values obtained (**a**) by Equation (11) of MD, (**b**) by the experimental interface TCR measurement and (**c**) by default value in Moldflow.

**Figure 14:**
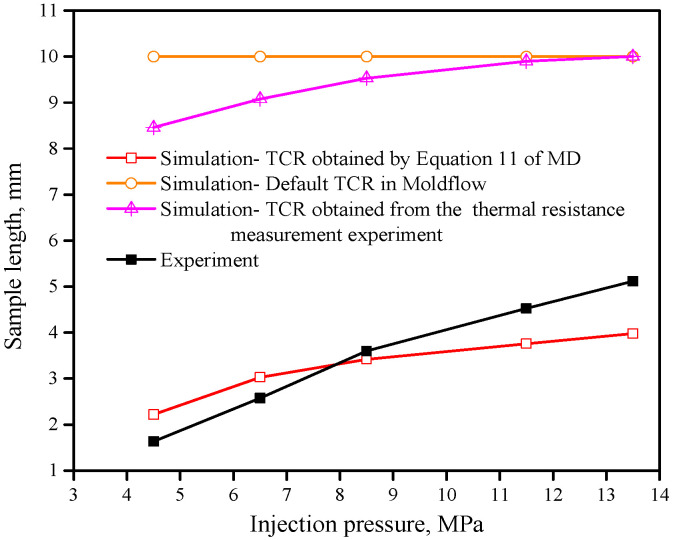
Injection sample length under different interface thermal resistance values and injection pressures.

The TCR values used in the micro-injection moulding simulation are shown in Table 2. Figure 13 shows the Moldflow simulation of injection sample length under different interface TCR values and injection pressures (the design sample length is 10 mm), and Figure 14 indicates the change curves of injection sample length under different TCR values and injection pressures. It can be seen that the actual sample length obtained by simulation using Moldflow’s default TCR value is the largest and does not change with an increase in injection pressure, and the error is also the largest compared with the actual injection experiment. The main reason for this finding is that Moldflow software does not consider the influence factors at the micro/nanoscale, such as wall slip and wettability. Because of Moldflow’s large TCR value, the degree of heat exchange between the polymer melt and the mould interface is small, and the heat loss is slow, so the melt can fill all spaces.

Using the TCR value measured by the thermal resistance experiment for the simulation, as the injection pressure increases, the simulated sample length increases from 8.46 to 10 mm, and the error compared with the actual injection experiment is also large; the maximum absolute error value reaches 6.83 mm (Figure 14). Using the TCR value calculated by Equation (10) of MD for the simulation, the sample length is between 2.22 and 3.98 mm under different injection pressures, which is closest to the corresponding sample length of the actual injection part. The maximum absolute error value is 1.13 mm, which is much smaller than the error of the sample length simulated by the TCR value measured by the thermal resistance experiment. Therefore, it can be considered that the TCR value obtained by the MD simulation better reflects the heat transfer performance of the micro-injection moulding. This is because the MD simulation TCR value takes into account the characteristics of the polymer molecular chain and its molecular chain morphology changes under different injection pressures. On the other hand, the heat conduction energy at the solid–liquid interface depends on the vibration of the phonons of the polymer material. The average free path of phonons of PMMA material is approximately 0.25 nm, and the flow channel width of the MD simulation is 5 nm, therefore the Knudsen number $k_{n}=0.05<0.1$, which satisfies the continuous flow characteristics. The TCR value of PMMA obtained by MD simulation does not exhibit a size effect, and the interface thermal resistance rule is adapted to the microscopic size.

Therefore, the method of TCR value calculated by MD simulation can more accurately and effectively reflect the heat conduction rule of the solid–liquid interface at the micro/nanoscale than that other methods, such as the thermal resistance measurement experiment and default TCR in Moldflow.

## 5. Conclusions

MD simulations were used to investigate the wall slip characteristics of PMMA polymer materials at the micro/nanoscale and their effects on the interface thermal resistance.

(1) When the mould wall is hydrophobic, the PMMA melt exhibits a significant wall slip phenomenon. As the external pressure increases, the wall slip speed and slip length increase. However, after a certain pressure is exceeded, the growth rate of the slip length is basically zero.
(2) As the external pressure increases, the PMMA molecular chains gradually start to separate, and the single molecular chain is untangled from the entangled grid. The chain breaks away from the wall after exceeding a certain threshold.
(3) Wall slip reduces the interface thermal resistance between the mould and the polymer melt and enhances the interface heat transfer performance. From the micro-injection moulding experiment results, it is found that the TCR value calculated by MD can more accurately reflect the heat conduction behaviour at the solid–liquid interface at the micro/nanoscale than that measured by the thermal resistance experiment, indicating that the micro/nano interface thermal resistance obtained by MD simulation is reliable. The established simulation model can be effectively used in the processing optimisation and die design for micro-injection industry.

## Figures and Tables

**Table 1:** Configuration parameters of the PMMA system.

Degree of Polymerisation	Number of Molecular Chains	Chemical Formula	Density (g/cm^3^)	System Size (nm)
100	20	C_5_H_8_O_2_	1.18	10 × 10 × 10

**Table 2:** TCR values in the micro-injection moulding simulation.

TCR Value (10^−5^ m^2^ K/W)	Injection Pressures (MPa)				
	4.5	6.5	8.5	11.5	13.5
By Equation (11) of MD	7.89	7.37	7.05	6.96	6.73
By the experimental interface TCR measurement	27.29	23.11	21.46	18.08	16.33
Default value in Moldflow	40	40	40	40	40
